# Training program-induced skeletal muscle adaptations in two men with myotonic dystrophy type 1

**DOI:** 10.1186/s13104-019-4554-z

**Published:** 2019-08-20

**Authors:** Marie-Pier Roussel, Marika Morin, Mélina Girardin, Anne-Marie Fortin, Mario Leone, Jean Mathieu, Cynthia Gagnon, Elise Duchesne

**Affiliations:** 1 Département des sciences fondamentales, Université du Québec à Chicoutimi, Saguenay, QC Canada; 2 Groupe de recherche interdisciplinaire sur les maladies neuromusculaires, Centre intégré universitaire de santé et de services sociaux du Saguenay–Lac-St-Jean, Installations de Jonquière, Saguenay, QC Canada; 3 Centre de recherche-Hôpital Charles-Le Moyne – Saguenay–Lac-Saint-Jean sur les innovations en santé, Saguenay, QC Canada; 4 Département des sciences de la santé, Université du Québec à Chicoutimi, Saguenay, QC Canada; 5 Faculté de médecine et des sciences de la santé, Université de Sherbrooke, Sherbrooke, QC Canada; 6 Unité d’enseignement en physiothérapie, Département des sciences de la santé, Université du Québec à Chicoutimi, 555, boulevard de l’Université, Saguenay, G7H 2B1 Quebec Canada

**Keywords:** Myotonic dystrophy type 1, Muscle plasticity, Training program, Muscle growth, Myogenesis, Leukocytes

## Abstract

**Objective:**

The purpose of this side product of another unpublished research project, was to address the effects of a training program on skeletal muscle adaptations of people with myotonic dystrophy type 1 (DM1), under a multifaceted perspective. The objective of this study was to look at training induced muscular adaptations by evaluating changes in muscle strength, myofiber cross-sectional area (CSA), proportion of myofiber types and with indirect markers of muscle growth [proportion of centrally nucleated fibers (CNF) and density of neutrophils and macrophages]. Two men with DM1 underwent a 12-week strength/endurance training program (18 sessions). Two muscle biopsies were obtained pre- and post-training program.

**Results:**

Muscular adaptations occurred only in Patient 1, who attended 72% of the training sessions compared to 39% for Patient 2. These adaptations included increase in the CSA of type I and II myofibers and changes in their proportion. No changes were observed in the percentage of CNF, infiltration of neutrophils and macrophages and muscle strength. These results illustrate the capacity of skeletal muscle cells to undergo adaptations linked to muscle growth in DM1 patients. Also, these adaptations seem to be dependent on the attendance.

*Trial registration* Clinicaltrials.gov NCT04001920 retrospectively registered on June 26th, 2019

## Introduction

Myotonic dystrophy type 1 (DM1), is the most frequent autosomal dominant myopathy in adults [1]. DM1 is caused by a cytosine, thymine and guanine (CTG) triplet repeat expansion within the dystrophy myotonic protein kinase gene [2]. The adult form of DM1 displays heterogeneous symptoms, namely skeletal muscle weakness. A maximal muscular strength decline from 24.5 to 52.8% on a 9 year period has been reported [3], in addition to loss of muscle mass [4–6], decrease of myogenic capacity [7–11] and perturbation of local and systemic inflammatory status [12, 13]. However, whether the loss of muscle mass is due to decreased protein synthesis or increased degradation is still unanswered [4, 6, 14, 15]. Muscle weakness secondary to muscle wasting is a strong predictor of disrupted social participation [16]. Research focusing on interventions that could induce positive skeletal muscle adaptations is much needed.

Strength-training is safe in patients with DM1 and may increase muscle strength [17]. However, it remains unknown if this gain can be explained by neuronal adaptations or muscle hypertrophy, which occurs when protein synthesis rate exceeds protein degradation. Protein synthesis is also influenced by the number of myonuclei present in skeletal muscle. Since exercise positively modulates satellite cell number [18], a complete evaluation of muscle hypertrophy should include myonuclei assessment. Moreover, exercise-induced muscle damage provokes an infiltration of macrophages and neutrophils, both cell types known to positively influence the myogenic process [19–24]. A scoping review has shown that the physiological parameters explaining muscle response to exercise are greatly understudied in DM1 [25]. The aim of this side product study of an unpublished project was to evaluate the effect of a strength and endurance-training program on skeletal muscle adaptation under a multifaceted perspective in two patients with DM1.

## Main text

### Methods

#### Participants

Participants recruited at the Neuromuscular Clinic of the *Centre intégré universitaire de santé et de services sociaux* (CIUSSS) of Saguenay–Lac-Saint-Jean (Québec, Canada) had to be between 20 and 60 years old, be able to walk without technical aid and have a muscle impairment rating scale (MIRS) grade 3 or 4 [26]. Exclusion criteria were any contraindication to physical exercise or to muscle biopsy. The study was approved by the Ethics Review Board of the CIUSSS Saguenay–Lac-St-Jean and a signed informed consent was obtained from each participant. The trial was retrospectively registered on June 26th, 2019 on Clinicaltrials.gov NCT04001920.

#### Training program

Participants underwent a 12-week/18-session supervised training program [17, 27] of 6 exercises: elbow flexion/extension, shoulder horizontal adduction, leg press, and knee flexion/extension. The one-repetition maximum (1-RM) was evaluated for each exercise at weeks 0 and 6. To offer a complete training program aimed at improving function it was divided: the first 6 weeks were dedicated to strength-training (2 sets of 6 repetitions at 80% of 1-RM), whereas the following weeks focused on endurance-training (1 set of 25 repetitions at 40% of 1-RM).

#### Muscle strength

The maximum isometric muscle strength of the knee extensors was assessed before and after the training program using make test with a handheld dynamometer (Microfet-2, Hoggan Health Industries, Salt Lake City, UT). The lever arm was measured to calculate the maximal torque in Newton-meters (Nm). Results were presented as the mean of the right and the left side.

#### Muscle biopsy

Suction-modified Bergström muscle biopsies were sampled in *Vastus lateralis* of each participant before and after the training program [28]. Two segments were taken from each biopsy and frozen separately in 2-methylbutane cooled in liquid nitrogen before being stored at − 80 °C. Four consecutive 10-μm-thick transverse sections were cut from each segment using a cryostat (CM1850; Leica Microsystems, Concord, Ontario, Canada). Each muscle biopsy provided a total of six sections from two distinct segments with two negative experimental controls. Two blinded evaluators analysed both segments to reinforce the results.

#### Immunohistochemical and histological analyses

Immunochemistry was performed using: CD68+ macrophages (Dako, Glostrup, Denmark), neutrophil elastase (Dako, Glostrup, Denmark) and anti-skeletal myosin fast (IIA, IIB and IIX isoforms) primary antibodies (Sigma-Adrich, St-Louis, MO, USA), biotinylated universal antibody anti-mouse IgG/rabbit IgG (Vector Laboratories, Burlingame, CA, USA) and chromogen AEC substrate (Dako, Glostrup, Denmark). A conventional haematoxylin/eosin staining was used to assess centrally nucleated fibers (CNF). Images of muscle sections were taken using AMG Evos XL Core Microscope. ImageJ Software (National Institues of Health, Bethesda, Maryland) was used to: (1) assess neutrophil and macrophage density (number of cells divided by muscle section volume), (2) identify fiber type (I or II) and measure their cross-sectional area (CSA) and (3) evaluate the proportion of CNF.

#### Statistical analysis

Statistical analyses were performed using Graphpad Prism version 7 (GraphPad Software, La Jolla, California). Pre- and post-training results of each patient compared to itself was performed with Student’s *t* tests. When the assumption of normality was not reached, the non-parametric Kruskal–Wallis Test was used. The significance level was set at p < 0.01.

### Results

#### Participants

The characteristics of the two patients are presented in Table 1. Despite the notable difference in the age and the body mass index (BMI), blood CTG repeat expansion size is equal and they both scored 4 on the MIRS [26]. A major difference can be observed in the training attendance: Patient 1 completed 72% (8/8 sessions in the first 6-week part, 5/10 in the second part) of the training program, whereas Patient 2 completed 39% of the program (2/8 sessions in the first part, 5/10 in the second part).

**Table 1:** Patients’ characteristics

# Patient	Age (years)	Height (m)	Weight (kg)	BMI (kg/m^2^)	CTG repeats	MIRS	Training attendance		Maximal isometric knee extensors strength (Nm)	
							(# of sessions)	(%)	Pre	Post
1	36	1.70	90.2	31.2	400	4	13/18	72	127.3	115.0
2	56	1.67	72.6	26.0	400	4	7/18	39	141.2	148.5

#### Skeletal muscle fiber size

**Fig. 1:**
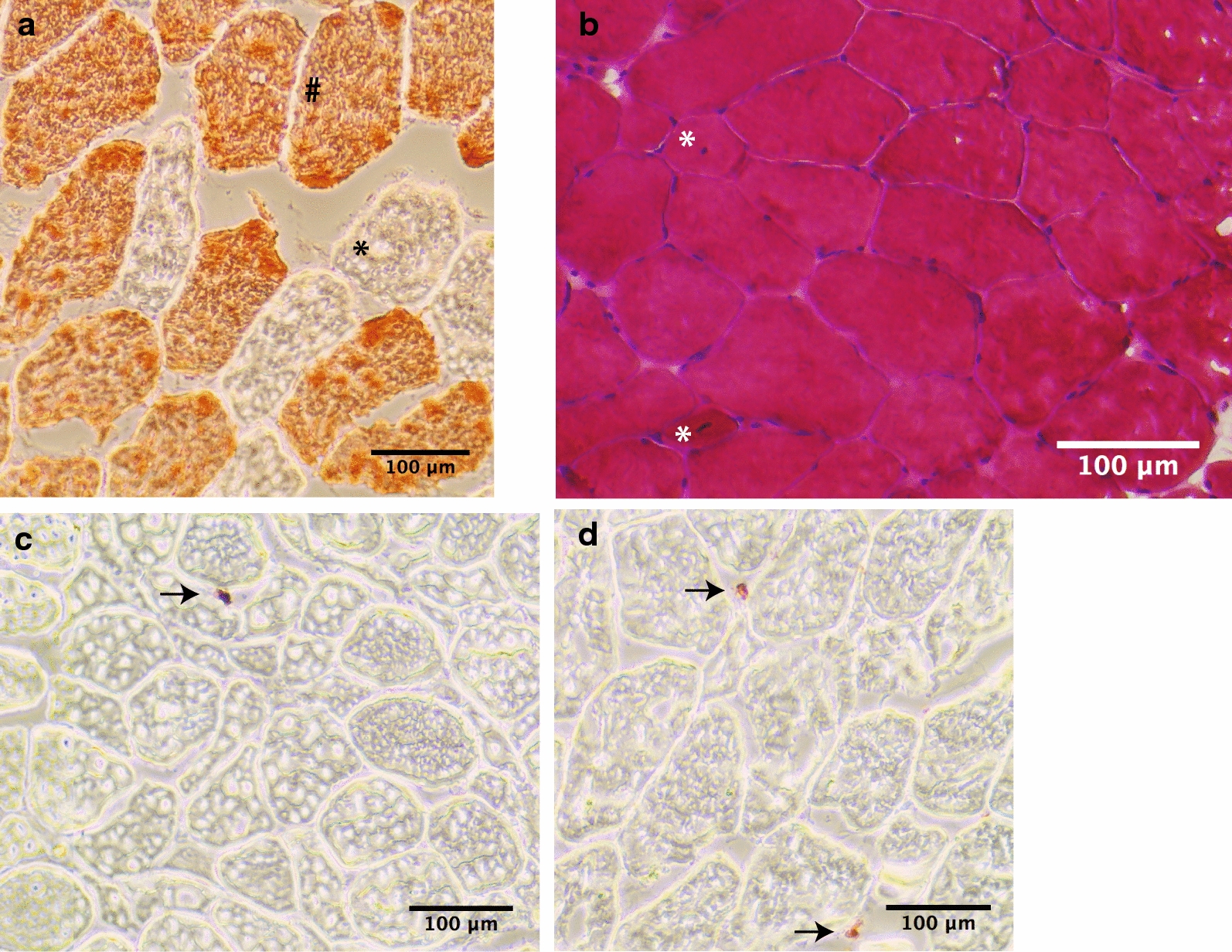
Histological analysis of *Vastus lateralis* muscle biopsies pre- and post-training program for DM1 patients. **a** Immunohistochemical staining of myofibers allowed the identification of fiber type II with anti-skeletal myosin fast primary antibody (#). Unstained cells correspond to fiber type I (*). **b** Haematoxylin/eosin allowed the identification of CNF (*). Immunochemistry allowed the identification of neutrophils (**c**) and macrophages (**d**), as indicated by the arrows

The immunohistochemical staining of myofibers is shown in Fig. 1a and results are presented in Table 2. For Patient 1, training induced a significant increase in CSA of type I myofibers reaching 38% and 28% for evaluators 1 and 2, respectively. To a lower extent, the same observations were made for type II myofibers CSA, but only evaluator 2 recorded a significant increase (20%). Contrasting results were seen for Patient 2 for whom the CSA of type I myofibers was not significantly different between the two time points while the size of type II myofibers significantly decreased according to both evaluators (by 16% and 14%).

**Table 2:** Histological and immunohistological results obtained in *Vastus lateralis* muscle biopsies pre- and post-training program for DM1 patients according to evaluator #1 and #2

	Evaluator #	Patient 1		Patient 2	
		Pre	Post	Pre	Post
CSA (type I), mm^2^ (SD)	1	0.0053 (0.00058)	0.0073 (0.0004)*	0.0061 (0.0008)	0.0052 (0.0016)
	2	0.0060 (0.001)	0.0077 (0.0003)*	0.0067 (0.0017)	0.0055 (0.0011)
CSA (type II), mm^2^ (SD)	1	0.0084 (0.0011)	0.0096 (0.0005)	0.0067 (0.0005)	0.0058 (0.0002)*
	2	0.0084 (0.0011)	0.0101 (0.0004)*	0.0067 (0.0005)	0.0059 (0.0004)*
Proportion (type I), % (SD)	1	57.3 (2.6)	28.2 (1.8)*	56.7 (5.8)	52.3 (3.0)
	2	56.6 (2.8)	28.7 (1.7)*	57.0 (4.4)	49.0 (7.6)*
Proportion (type II), % (SD)	1	42.7 (2.6)	71.8 (1.8)*	43.3 (5.8)	47.7 (3.0)
	2	43.4 (2.8)	71.3 (1.7)*	43.0 (4.4)	51.0 (7.6)*
CNF proportion % (SD)	1	4.77 (3.4)	5.28 (3.4)	7.14 (6.3)	8.26 (2.9)
	2	2.18 (2.4)	2.08 (1.7)	6.3 (5.4)	4.45 (1.5)
Neutrophil density, cells/mm^3^ (SD)	1	218 (113)	353 (125)	445 (237)	356 (164)
	2	445 (286)	519 (244)	633 (168)	429 (170)
Macrophage density, cells/mm^3^ (SD)	1	2852 (413)	2642 (371)	4347 (803)	5158 (972)
	2	3855 (669)	4273 (979)	5846 (1555)	7830 (501)*

*Significant difference between pre- and post-training (p < 0.01)

#### Proportion of muscle fiber type

As presented in Table 2, significant change in muscle fiber composition is observed in Patient 1 following its participation to the training program: an increase of 68% and 64% of type II myofiber was reported by evaluator 1 and 2, respectively, with a concomitant decrease in the proportion of type I fibers. On the other hand, while evaluator 1 reported no significant impact of training program on muscle fiber type composition for Patient 2, evaluator 2 noted a slight but significant increase in the proportion of type II fiber post-training (19%).

#### Proportion of CNF and leukocyte accumulation

The proportion of CNF was evaluated as a measure of myogenic activity (Fig. 1b) along with the density of neutrophil and macrophage (Fig. 1c, d), known as myogenic regulators. Following the training program, both evaluators reported no significant difference in the proportion of CNF nor neutrophil and macrophage densities for both patients, except for evaluator 2 which concluded to a significant increase of 34% in macrophage density for Patient 2 (Table 2).

### Discussion

Training programs seem a promising strategy to slow or reverse muscle atrophy in DM1, but there is no clear evidence if they could trigger cellular and molecular responses similar to the ones observed in healthy individuals. The main findings of this paper are that skeletal muscle can undergo growth adaptations.

It is interesting to note that fiber CSA expanded in one participant despite that the training program did not induce any change in maximal strength. A non-linear relationship between maximal force and CSA has already been described and explained by the other factors such as age, gender and training status [29]. Furthermore, a single isometric muscle strength evaluation carried out at the end of the training program, which was different from isokinetic exercises performed during the training program, could have not reflected the positive fundamental muscle adaptations since motor control is a major component implicated in the assessment of muscle strength.

Tollbäck et al. [30] have reported no significant difference in average fiber size in patients with DM1 that participated in their supervised 12-week progressive high-resistance training program. Our results demonstrated that the training program has only induced a significant increase in myofiber CSA (type I and II) of Patient 1, who completed 72% of the training program, including all strength-training sessions. Patient 2, who attended only 39% of the training sessions (and only 25% of the strength part) has shown a slight, but significant, decrease in type II myofiber CSA. Unsurprisingly, these results suggest that the intensity of the stimulus is an important factor in muscle growth. The proportion of type II muscle fibers has significantly increased in Patient 1 and to a much lesser extent for only one evaluator for Patient 2. Since the training program was divided in two 6-week periods dedicated to strength-training and endurance-training respectively, the adaptations observed could have been flattened.

Protein synthesis-induced muscle growth is influenced by myogenic activity and inflammatory cell invasion: satellite cells are guided through the different phases of myogenesis by the activity of inflammatory cells [19, 23, 24, 31]. Regenerating fibers are characterized by their small caliber and their centrally located myonuclei [32]. While the presence of CNF is generally considered as an indicator of myogenesis in studies focusing on skeletal muscle repair, it represents a sign of histological deterioration in the neuromuscular disease field. This is because some muscular dystrophies (e.g. Duchenne muscular dystrophy) are characterized by repeated cycles of degeneration-regeneration leading to an abnormally large number of CNF. Strength training has previously induced no systematic difference in histopathological abnormalities in DM1 [30], the proportion of CNF could then be considered as a myogenesis marker in people with DM1 that have undergone training. In this study, no significant differences were observed in the proportion of CNF in both patients included in this study. While reinforcing the previous findings that training is safe for patients with DM1, our results suggest that either myogenic process was not triggered by this training stimulus or either this process has taken place before the sampling of muscle biopsy post-training in our participants.

Finally, in our participants, neutrophil and macrophage densities were not significantly modulated by the training program: only evaluator 2 has reported a significant increase in macrophage density post-training for Patient 2. It was not surprising that neutrophil density did not change since, beyond their pro-inflammatory role, neutrophils participate in the early stages of the myogenic process which typically lasts 4–5 days [24]. Conversely, while macrophages are classically known for their pro-inflammatory roles in innate immunity, a subset of macrophages, called M2, participate in the repair and remodeling processes. Since the antibody used in this study was pan-macrophage, the subset(s) of macrophage which has undergone an increase in Patient 2 (by evaluator 2) cannot be identified.

Our results suggest that muscular adaptations linked to muscle growth can occur in DM1 as demonstrated by the CSA increase of type I and type II myofibers. Training might also influence the distribution of myofibers, in favour of type II. The myogenic and inflammatory markers evaluated do not seem to be modulated by the training stimulus in our participants. Compliance to the program seems to be an important factor to consider. Patient’s preferences regarding training regimen should be considered in the perspective of personalized training/precision medicine. It should be noted that beyond the positive impact of training on muscle adaptations, it could also bring positive changes in other organ systems. Further studies comprising a higher number of participants and controls are needed to validate our findings and determine to which extent and how skeletal muscles of DM1 patients adapt to strength training.

## Limitations

- This study only has two participants, which limits generalizations to the whole DM1 population.
- The two patients included in this study cannot be directly compared together considering the difference in training attendance, age and BMI.
- This project serves as a template for further studies with more DM1 participants regarding post-training biopsy evaluations.

## Data Availability

The datasets used and/or analysed during the current study are available from the corresponding author on reasonable request.

## Abbreviations

BMI: body mass index
CIUSSS: Centre intégré universitaire de santé et de services sociaux
CNF: centrally nucleated fibers
CSA: cross-sectional area
CTG: cytosine, thymine and guanine triplet
DM1: myotonic dystrophy type 1
MIRS: muscle impairment rating scale
